# Prostaglandin E_2_ in tick saliva regulates macrophage cell migration and cytokine profile

**DOI:** 10.1186/1756-3305-6-261

**Published:** 2013-09-11

**Authors:** Nina M Poole, Gayatri Mamidanna, Richard A Smith, Lewis B Coons, Judith A Cole

**Affiliations:** 1 Department of Biological Sciences, The University of Memphis, 239 Ellington Hall, 3700 Walker Avenue, Memphis TN, 38152, USA; 2 Department of Orthopaedic Surgery, The University of Tennessee-Campbell Clinic, Suite 520, 1211 Union Avenue, Memphis TN, 38104, USA; 3 Department of Molecular Virology and Microbiology, Baylor College of Medicine, One Baylor Plaza, Houston 77047, TX, USA

**Keywords:** Tick, *Dermacentor variabilis*, Saliva, PGE_2_, Macrophage, Migration, Cytokines

## Abstract

**Background:**

Ticks are obligate hematophagous ectoparasites that suppress the host’s immune and inflammatory responses by secreting immuno-modulatory and anti-inflammatory molecules in their saliva. In previous studies we have shown that tick salivary gland extract (SGE) and saliva from *Dermacentor variabilis* have distinct effects on platelet-derived growth factor (PDGF)-stimulated IC-21 macrophage and NIH3T3-L1 fibroblast migration. Since tick saliva contains a high concentration of prostaglandin E_2_ (PGE_2_), a potent modulator of inflammation, we used a PGE_2_ receptor antagonist to evaluate the role of PGE_2_ in the different migratory responses induced by saliva and its impact on macrophage cytokine profile.

**Methods:**

Adult ticks were fed on female New Zealand white rabbits for 5-8 days. Female ticks were stimulated with dopamine/theophylline to induce salivation and saliva was pooled. Competitive enzyme immunoassays (EIA) were used to measure saliva PGE_2_ content and the changes in macrophage intracellular cyclic adenosine monophosphate (cAMP) levels. The effects of tick saliva on macrophage and fibroblast migration were assessed in the absence and presence of the PGE_2_ receptor antagonist, AH 6809, using blind well chamber assays. A cytokine antibody array was used to examine the effects of tick saliva on macrophage cytokine secretion. Statistical significance was determined by one-way ANOVA; Student Newman-Kuels post-test was used for multiple comparisons.

**Results:**

The saliva-induced increase in PDGF-stimulated macrophage migration was reversed by AH 6809. The inhibition of PDGF-stimulated fibroblast migration by saliva was also antagonist-sensitive. Tick saliva induced macrophages to secrete copious amounts of PGE_2_, and conditioned medium from these cells caused an AH 6809-sensitive inhibition of stimulated fibroblast migration, showing that macrophages can regulate fibroblast activity. We show that tick saliva decreased the secretion of the pro-inflammatory cytokines regulated and normal T cell expressed and secreted (RANTES/CCL5), tumor necrosis factor-alpha (TNF-α), and soluble TNF receptor I (sTNFRI) through a PGE_2_-dependent mechanism mediated by cAMP. Saliva had similar effects on lipopolysaccharide (LPS) stimulated macrophages.

**Conclusions:**

Our data show that ticks utilize salivary PGE_2_ to subvert the ability of macrophages to secrete pro-inflammatory mediators and recruit fibroblasts to the feeding lesion, therefore inhibiting wound healing.

## Background

Ixodid ticks such as *Dermacentor variabilis* are obligate blood-sucking ectoparasites that physically attach to their host for several days to feed until repletion. The cutting action of the chelicerae, insertion of hypostome, and the rupturing of blood vessels [1-3] all result in localized damage to the host’s epidermis and dermis. This mechanical damage to the host’s skin should elicit the host’s immune, inflammatory, hemostatic, and wound healing responses resulting in removal or rejection of the tick; but this is not the case. Instead, ticks use a cocktail of bioactive molecules in their saliva to evade these host responses [4-12].

Tick saliva has been shown to regulate the migratory activities of different cell types by modulating cell signaling [13-15] and the activity of chemokine binding proteins [16-21]. Tick salivary constituent(s) have suppressive effects on innate immunity by regulating neutrophil recruitment [22], adherence [23], phagocytosis [24], and apoptosis [25] and natural killer cell activity [26,27]. In antigen-presenting cells, saliva reduces macrophage cytokine production [28,29], co-stimulatory molecule expression [28,30], phagocytosis [14], and nitric oxide production [26] and inhibits dendritic cell differentiation, maturation, and cytokine production [31-33]. Tick saliva also contains molecules that control host angiogenesis and wound healing to aid feeding [34-38].

Prostaglandins are among the most abundant bioactive molecules in tick saliva reviewed in [39]. Prostaglandin E_2_ (PGE_2_), which increases vasodilation [40] and decreases inflammation by regulating cytokine production [41-45], is found in high concentration in tick saliva [39,46-50]. The exact role(s) of prostaglandins in tick saliva have not all been identified but it has been shown that salivary PGE_2_ inhibits dendritic cell differentiation, maturation, and cytokine production [31,32] and T lymphocyte proliferation [47].

We have previously demonstrated that tick salivary gland extract (SGE) and saliva have distinct effects on platelet-derived growth factor (PDGF)-stimulated fibroblast [15] and macrophage [14] migration. PGE_2_ has been shown to regulate the migratory activities of these cells [51-54]. Therefore, in this study we use IC-21 macrophages and NIH3T3-L1 fibroblasts to determine if the PGE_2_ found in *D. variabilis* saliva can mimic this regulation and is responsible for the different migratory responses induced by saliva previously noted by using the PGE_2_ receptor antagonist AH 6809. Since the cytokines secreted by macrophages regulate the inflammatory and cellular immune responses during wound healing, we also used this approach in evaluating the effects of salivary PGE_2_ on macrophage cytokine secretion.

## Methods

### Cell culture

Depending on the life stage, *D. variabilis* can feed on a variety of hosts ranging from small rodents to larger mammals such as humans. For this study, IC-21 murine peritoneal macrophages were used because they are a continuous monoclonal murine macrophage-like cell line very similar to macrophages in morphology [55], phagocytic and cytolytic activities [56], expression of platelet-activating factor receptors [57], and can be activated by lipopolysaccharide (LPS) via Toll-like receptor 4 (TLR4) [55]. Macrophages were maintained in 25 cm^2^ flasks or 100 mm dishes in RPMI 1640 (MediaTech, Herndon, VA) supplemented with 10% fetal bovine serum (FBS), 100 U/ml penicillin and 100 μg/ml streptomycin. They were subcultured weekly using Ca^2+^/Mg^2+^ free Hanks balanced salt solution (HBSS) (Mediatech, Herndon, VA) and seeded at a density of 5 × 10^4^ or 6 × 10^4^ cells/ml. NIH3T3-L1 murine dermal fibroblasts, a common fibroblast model, were grown in 25 or 75 cm^2^ flasks in Dulbecco’s modified Eagle’s medium nutrient mixture F-12 (DMEM/F12) (MediaTech, Herndon, VA) supplemented with 10% FBS, 100 U/ml penicillin and 100 μg/ml streptomycin, and were subcultured weekly using Ca^2+^/Mg^2+^ free HBSS and 0.025% trypsin/0.02% EDTA (Mediatech, Herndon, VA) then seeded at a density of 5 × 10^4^ cells/ml.

### Collection of tick saliva

Adult male and female ticks were purchased from Etco Services, Inc (Henderson, NC) and maintained in 96% humidity with a saturated K_2_SO_4_ solution at room temperature. Ixodid tick feeding occurs in two phases: slow feeding and rapid feeding [3]. In adult ixodid females, slow feeding lasts 6 or more days with a 10-fold weight gain, and it is during this time salivary constituents important to the tick’s ability to survive on the host are more likely to be present in high concentration in the saliva [58]. The rapid feeding phase is 12–24 hours before engorgement is reached in which body weight increases another 10-fold [59]. Therefore, ticks were fed on adult female New Zealand white rabbits (Harlan Laboratories, Prattville, AL) for 5–8 days (slow feeding stage) following protocols approved by The University of Memphis Institutional Animal Care and Use Committee. Partially engorged females (70–350 mg) were removed and attached to a microscope slide with double-sided adhesive tape. Female ticks were injected with 10 μl of MOPS buffered tick saline (pH 7.0) containing 10 mM dopamine / 10 mM theophylline with 3% dimethyl sulfoxide (DMSO) [60]. Ticks that did not salivate 5 min post-injection were not used. Ticks salivating were injected a total of 3 times in 5 min intervals and saliva was collected in a 25 μl non-heparinized soda lime glass micropipette, kept on ice, and pooled. Total protein concentration of saliva was determined using a Bio-Rad Protein Assay based on the method of Bradford (Bio Rad Laboratories, Hercules, CA) and stored at −20°C until used. Since the majority of the tick salivary components identified are proteins [9], we reported saliva used in μg protein/ ml.

### PGE_2_ measurement

To determine the amount of PGE_2_ in *D. variabilis* saliva and how saliva affects the amount of PGE_2_ secreted by macrophages, a competitive PGE_2_ Enzyme Immunoassay (EIA) Express kit (Cayman Chemical, Ann Arbor, MI) was used. Macrophages were cultured at a density of 5 × 10^4^ cells/well in 24-well plates for 5 days and changed to medium containing 2% FBS 24 h prior to the experiment. Cells were treated with vehicle phosphate buffered saline (PBS) or saliva (1.2 or 3.6 μg protein/ml) for 18 h [61]. The conditioned medium was collected then stored at −80°C and PGE_2_ content was measured according to manufacturer’s instructions. Absorbance was read at 405 nm using a Bio-Tek Elx808 Ultra Microplate Reader. A standard curve linearized using a logit transformation and a linear regression fit was used to determine PGE_2_ concentrations.

### Cell migration assay

The effects of salivary PGE_2_ on macrophage and fibroblast migration were assessed using blind well chemotaxis chamber assays (Neuro Probe, Gaithersburg, MD). Macrophages grown to confluence in 100 mm dishes were incubated for 15 min with Ca^2+/^Mg^2+^ free HBSS. Cells were removed from the surface by pipetting and then resuspended in serum-free medium, counted, and diluted to a concentration of 1 × 10^5^ cells/ml. Confluent fibroblasts were removed from flasks by incubation for 15 min with Ca^2+/^Mg^2+^ free HBSS and trypsinization for 5 min. Cells were also resuspended in serum-free medium, counted, and diluted to a concentration of 1 × 10^5^ cells/ml. The lower chamber of the blind well (Neuro Probe, Gaithersburg, MD) was loaded with either serum-free medium or medium with 100 ng/ml platelet-derived growth factor (isoform PDGF-BB homodimer) (ProSpec-Tany TechnoGene Ltd, East Brunswick, NJ) as the chemoattractant. An 8 μm uncoated polycarbonate filter (Neuro Probe, Gaithersburg, MD) was placed between the lower and upper chambers of each blind well. The upper chamber was loaded with 100 μl of the macrophage suspension pretreated for 30 min with vehicle (DMSO), saliva (2.4 μg protein/ml), or PGE_2_ (1 μM; Cayman Chemical, Ann Arbor, MI) in the absence or presence of the E and D prostanoid (EP and DP) receptor antagonist AH 6809 (10 μM; Cayman Chemical, Ann Arbor, MI). For the fibroblast suspensions, 30 min pretreatments consisted of vehicle (DMSO), saliva (2.4 μg protein/ml), PGE_2_ (1 μM), or conditioned medium (CM) from macrophages treated with saliva (2.4 μg protein/ml) for 18 h (to allow PGE_2_ to accumulate) in the absence or presence of AH 6809. The blind wells were incubated for 4 h at 37°C in humidified air with 5% CO_2_. After the incubation period, the non-invading cells were removed from the upper surface of the filters with a cotton-tip applicator. The filters were placed upside-down on a microscope slide and the cells were fixed with 100% methanol, stained with 0.4% crystal violet in 4% ethanol, and counted in five random high-power (40x) fields using a Nikon Labophot light microscope (Nikon, Melville, NY). Data were reported as the percentage of control cells migrating in 4 h.

### Cytokine array

To evaluate the effects of PGE_2_ in the saliva-induced changes on macrophage cytokine secretion we used the RayBio® Mouse Cytokine Antibody Array (Catalog # AAM-CYT-1-8) RayBiotech, Inc., Norcross, GA), which simultaneously detects 22 cytokines. Macrophages were cultured at a density of 5 × 10^4^ cells/ml in 6-well plates and grown to confluence. Twenty-four hours prior to the experiment, cells were changed to medium containing 2% FBS. Cells were treated with vehicle (DMSO), saliva (2.4 μg protein/ml), 10 μM AH 6809, saliva + AH 6809, 0.76 μg/ml lipopolysaccharide (LPS) (InvivoGen, San Diego, CA), or saliva + LPS for 18 h [61]. LPS is a Gram-negative bacteria toxin which activates macrophages through Toll-like receptor 4 (TLR4). The conditioned medium was collected, and the cytokine content for each sample was determined according to manufacturer’s instructions. Arrays were developed with kit detection buffer and exposed to Classic Blue Autoradiography Film X (Molecular Technologies, St. Louis, MO) for 0.5, 2.5, 1, and 5 min. The intensities of signals for each cytokine were quantified by densitometry using ImageJ version 1.46 Windows (National Institutes of Health, Bethesda, MD, http://rsb.info.nih.gov/ij/). The vehicle-treated array was used as the reference array to which the signals of the other arrays were normalized. Data were reported as relative expression levels for each exposure time.

### Mouse interleukin-1 beta (IL-1β) enzyme-linked immunosorbent assay (ELISA)

To evaluate the effects of saliva on IL-1β secretion, we used a RayBio® Mouse IL-1β ELISA Kit (Catalog # ELM-IL1beta-001 RayBiotech, Inc, Norcross, GA). Macrophages were cultured at a density of 5 × 10^4^ cells/well in 24-well plates and grown until confluent. The cells were changed to medium containing 2% FBS 24 h prior to the experiment. Macrophages were treated with vehicle (PBS) or saliva (1.2 or 3.6 μg protein/ml) and then stimulated for 18 h with 0.76 μg/ml LPS [61]. The cells were then pulsed with 5 mM adenosine triphosphate (ATP) (activator of purinergic receptor P2X7) for 20 min and cultured for an additional 3 h. The conditioned medium was collected then stored at −80°C and IL-1β content was measured according to manufacturer’s instructions. Absorbance was read at 450 nm using a Bio-Tek Elx808 Ultra Microplate Reader; data were normalized to the absorbance in controls and reported as the fold change in IL-1β secretion.

### Cyclic adenosine monophosphate (cAMP) measurement

A cAMP competitive EIA assay kit (Cayman Chemical, Ann Arbor, MI) was utilized to determine intracellular cAMP concentrations. Macrophages were cultured at a density of 5 × 10^4^ cells/well in 24-well plates for 5 days. Cells were washed with serum-free medium and then cultured in RPMI with 0.5 mM 3-Isobutyl-1-methylxanthine (IBMX) (Sigma-Aldrich, St. Louis, MO) for 30 min. Macrophages were then pre-treated with AH 6809 for 15 min and challenged with vehicle (DMSO), saliva (1.2 and 3.6 μg protein/ml), or 3 μM PGE_2_ for 7.5 min. Cells were lysed in 0.1 M HCL at room temperature for 20 min and dissociated by pipetting, and samples were collected and centrifuged at 1000 xg for 10 min at room temperature. Cyclic AMP determination was performed according to manufacturer’s instructions. Absorbance was read at 405 nm using a Bio-Tek Elx808 Ultra Microplate Reader, and a standard curve was linearized using a logit transformation and a linear regression fit was used to determine cAMP concentrations.

### Statistical analysis

PGE_2_ and cAMP data are means ± standard errors of means (SEM) of 3 experiments assayed in duplicate performed over several passages of cells. Cell migration data are a percentage of control values ± SEM of 3 experiments. Data for the cytokine array are presented as means ± SEM normalized to the vehicle treated array and reported as relative expression levels determined by densitometry for 2 exposure times. Statistical significance was determined by one-way ANOVA; Student Newman-Kuels post test was used for multiple comparisons employing Graph Pad Prism version 3.02 Windows (Graph Pad Software, San Diego CA, http://www.graphpad.com). Differences in means were considered significant at p ≤ 0.05.

## Results

### Tick saliva increases macrophage PGE_2_ secretion

**Figure 1:**
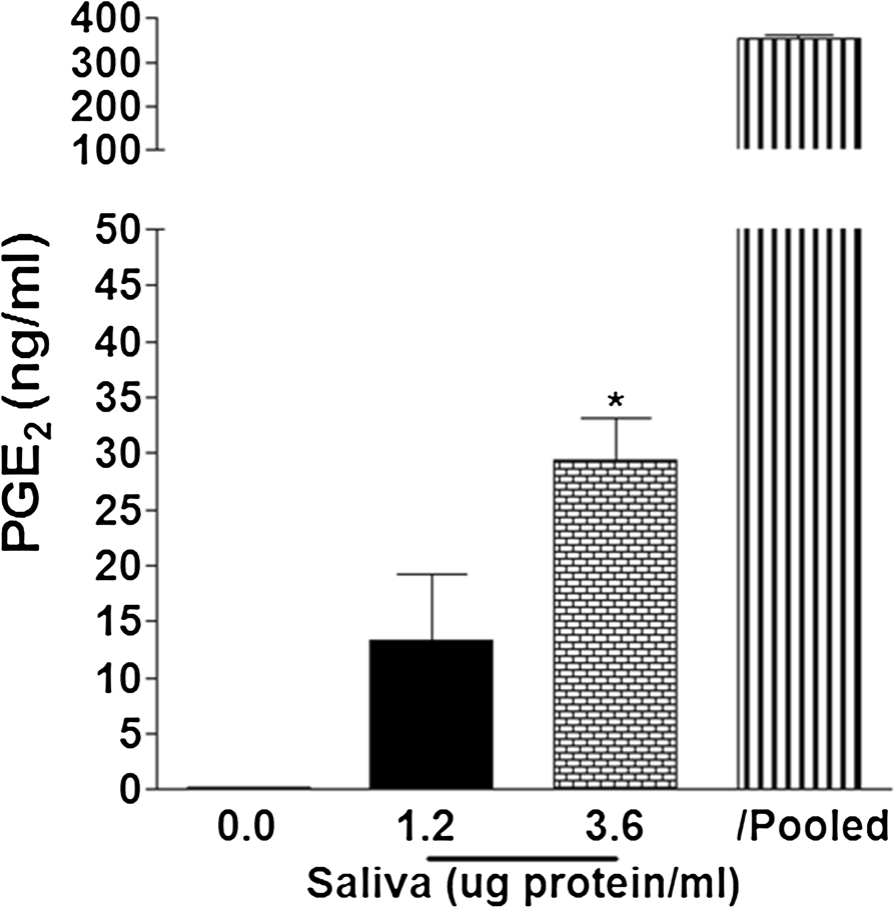
**Tick saliva causes PGE**_**2 **_**secretion by macrophages.** Cells were treated with vehicle (PBS) or saliva for 18 h. Increasing the dose of saliva resulted in a higher level of macrophage PGE_2_ secretion reaching significance (p < 0.05) at 3.6 μg protein/ml, (*) p < 0.01 when compared to vehicle control. The PGE_2_ concentration of the pooled saliva used for all experiments was 352 ± 9 ng/ml, which was diluted to deliver 3.6 μg protein/ml. If still present in the sample, the PGE_2_ from the pooled saliva would only account for approximately 1.2% of the total PGE_2_. Data are means ± SEM, n = 3 assayed in duplicate.

PGE_2_ is one of the most important prostanoids that plays a role in both anti- and pro-inflammatory responses. We used a PGE_2_ EIA assay to measure the effects of tick saliva on macrophage PGE_2_ secretion. Cells were treated for 18 h with vehicle (PBS) or saliva (1.2 or 3.6 μg protein/ml). Increasing the dose of saliva induced a significantly higher level of macrophage PGE_2_ secretion. Saliva (3.6 μg protein/ml) increased macrophage secretion of PGE_2_ from 0.1 ± 0.04 to 29 ± 4 ng/ml (Figure 1). Since PGE_2_ is found in the saliva of many tick species [31,32,39,46-50], we determined that the total PGE_2_ concentration of the pooled *D. variabilis* saliva used in these experiments was 352 ± 9 ng/ml (Figure 1). We used 12 μl/ml of the pooled saliva to deliver 3.6 μg protein/ml. Therefore, if the PGE_2_ from the tick saliva is still present in our sample after 18 h, it only accounts for approximately 1.2% of the total PGE_2_ in the sample.

### Salivary PGE_2_ regulates macrophage and fibroblast migration

**Figure 2:**
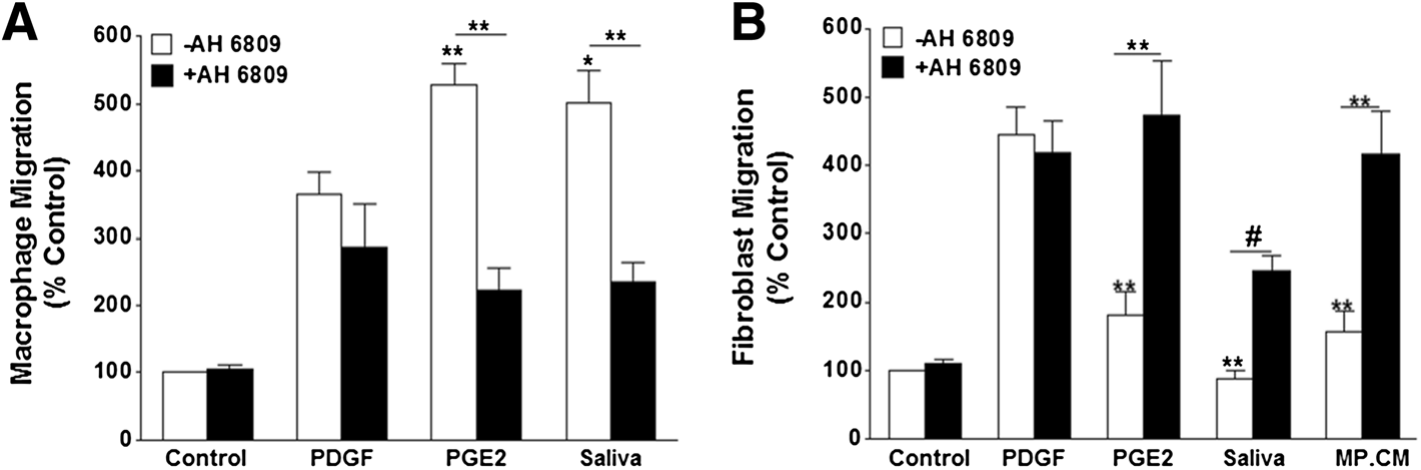
**Saliva-induced effects on macrophage and fibroblast migration are PGE**_**2 **_**receptor antagonist-sensitive. (A)** Macrophages were pretreated for 30 min with vehicle (DMSO), saliva (2.4 μg protein/ml), or PGE_2_ (1 μM) in the absence or presence of the PGE_2_ receptor antagonist AH 6809 (10 μM), and then incubated for 4 h. PDGF increased the number of cells migrating, an effect enhanced by saliva and PGE_2,_ (**) p < 0.001 and (*) p < 0.01 when compared to PDGF treatment only. These effects were reversed by AH 6809, (**) p < 0.001 when saliva treatment was compared to saliva + AH 6809 and when PGE_2_ was compared to PGE_2_ + AH 6809. **(B)** Fibroblasts were pretreated for 30 min with vehicle (DMSO), saliva (2.4 μg protein/ml), PGE_2_ (1 μM), or conditioned medium from macrophages treated with saliva for 18 h in the absence or presence of the AH 6809 (10 μM) using PDGF as the chemoattractant. After 4 h, saliva, PGE_2_, and saliva-treated macrophage conditioned medium decreased fibroblast migration (CM), (**) p < 0.001 when compared to PDGF treatment only. The effects of saliva were partially reversed by AH 6809; however, the receptor antagonist fully restored the migration of cells treated with conditioned medium of saliva-treated macrophages (CM) similar to that of PGE_2_ in the presence of AH 6809, (**) p < 0.001 when PGE_2_ was compared to PGE_2_ + AH 6809 and when CM was compared to CM + AH 6809, (#) p < 0.05 when saliva was compared to saliva + AH 6809. Data are reported as the % control values and are means ± SEM, n = 3.

To determine the role of PGE_2_ in the effects of saliva on macrophage migration, we utilized blind well chemotaxis chamber assays. Cells were pretreated with vehicle (DMSO), saliva (2.4 μg protein/ml), or 1 μM PGE_2_ in the absence or presence of 10 μM AH 6809 for 30 min then loaded into the upper chamber. The lower chamber was loaded with medium for basal migration or medium containing 100 ng/ml PDGF for stimulated migration. PDGF increased the total number of cells migrating by 264 ± 33%, an effect enhanced by saliva consistent with previous observations in our laboratory [14] (Figure 2A). The saliva-induced increase in PDGF-stimulated macrophage migration was similar to the increase induced by PGE_2_ (Figure 2A). The PGE_2_ receptor antagonist AH 6809 significantly reduced the stimulatory effects of saliva by 53 ± 30% (Figure 2A). This reduction was similar to the effects observed in cells treated with PGE_2_ in the presence of the receptor antagonist 58 ± 32% (Figure 2A) which suggests the saliva-induced increase in macrophage migration was mediated by PGE_2_.

Fibroblast migration is inhibited by PGE_2_[52-54], saliva [13], and SGE [15]. Therefore, we treated fibroblasts with saliva (2.4 μg protein/ml) in the presence or absence of 10 μM AH 6809 for 4 h to determine if the PGE_2_ content in *D. variabilis* saliva is responsible for this inhibition. We also treated these cells with conditioned medium from macrophages treated with saliva (2.4 μg protein/ml) for 18 h, since saliva induces macrophages to secrete substantial amounts of PGE_2_ (Figure 1). The number of fibroblasts migrating in response to PDGF 346 ± 40% was significantly reduced by saliva to 88 ± 11% and conditioned medium from saliva-treated macrophages to 156 ± 31% (Figure 2B). The inhibitory effects of saliva and macrophage conditioned medium were similar to that of PGE_2_ and were antagonized by AH 6809 (Figure 2B). This antagonism partially restored the migration of the saliva-treated cells 55 ± 17% while there was full restoration in the cells treated with conditioned medium from saliva-treated macrophages 94 ± 21% when compared to the PDGF-stimulated cells (Figure 2B). These effects indicate PGE_2_, at least in part, was responsible for the inhibition (Figure 2B).

### Tick saliva decreases macrophage cytokine secretion, a response sensitive to the PGE_2_ receptor antagonist AH 6809

**Figure 3:**
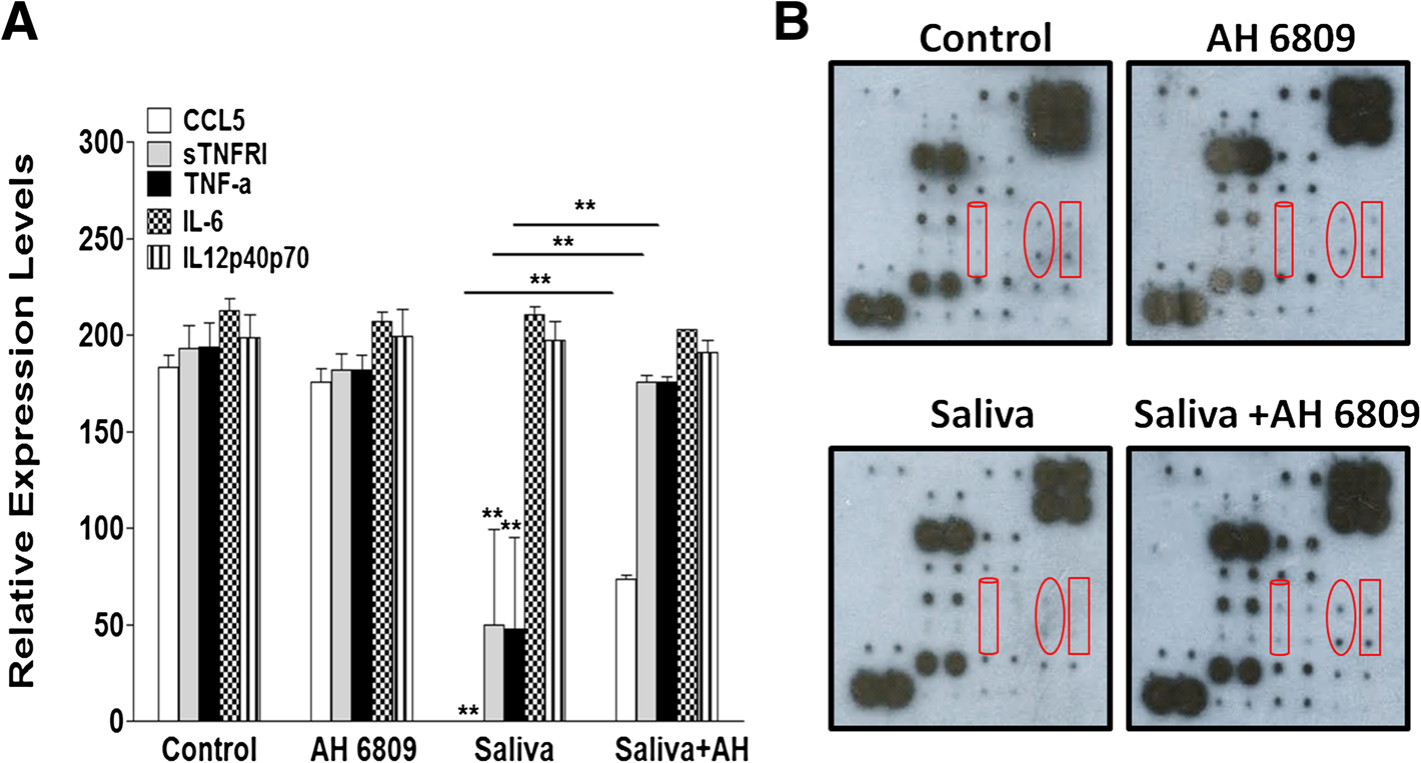
**Saliva-induced decreases on CCL5, sTNFRI, and TNF-α secretion by macrophages are PGE**_**2 **_**receptor antagonist-sensitive.** Cells were treated with vehicle (DMSO), saliva (2.4 μg protein/ml), AH 6809 (10 μM), or saliva + AH 6809 for 18 h. **(A)** Saliva reduced the relative expression levels of secreted CCL5, soluble TNF Receptor I (sTNFRI), and TNF-α which was reversed by AH 6809. **(B)** Image of blots exposed to film for 2.5 min corresponding to the treatments in **(A)**, cylinder = CCL5, oval = sTNFRI, and rectangle = TNF-α, (**) p < 0.001 when compared to vehicle control and when saliva was compared to saliva + AH 6809. Data are means ± SEM normalized to the vehicle treated array reported as relative expression levels determined by densitometry for 1 and 2.5 min exposure times.

**Figure 4:**
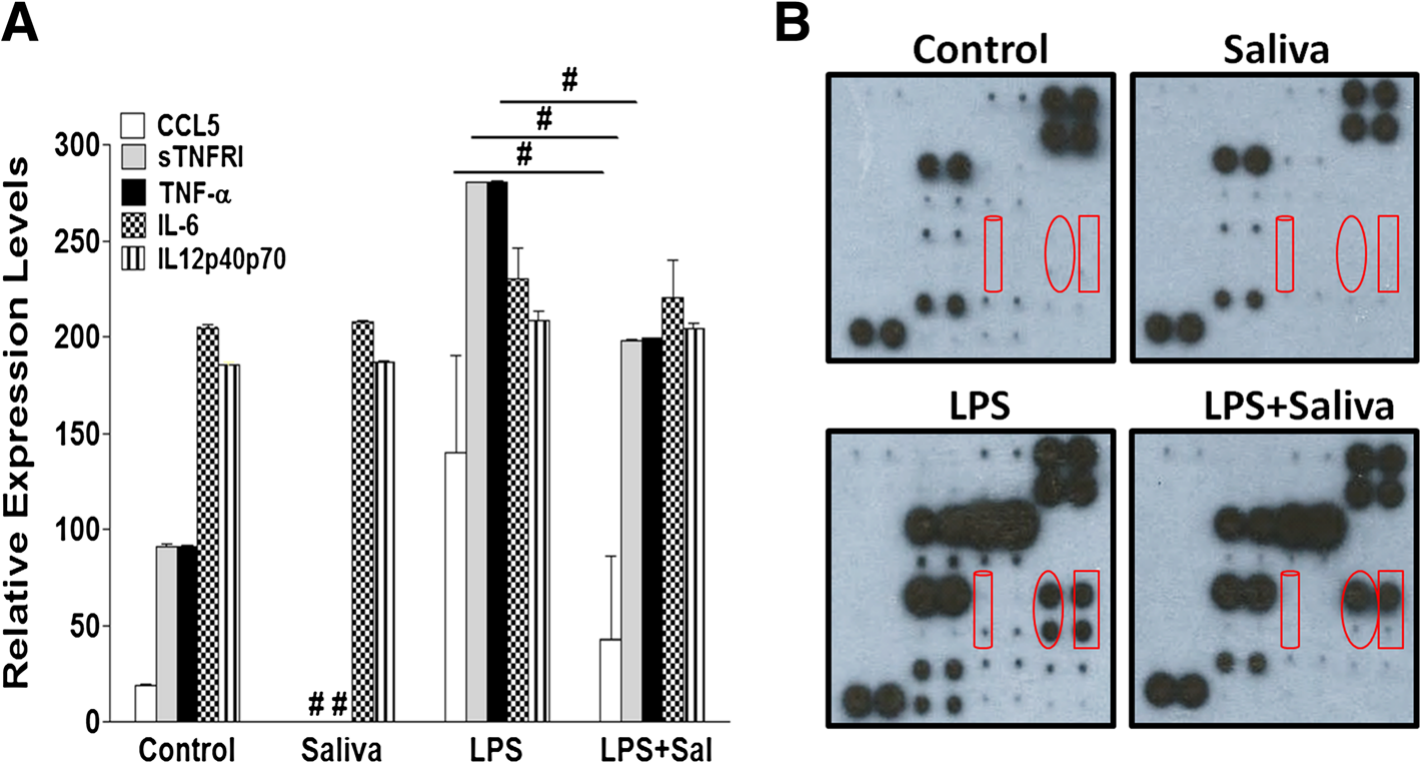
**Saliva reduces LPS-stimulated cytokine secretion by macrophages.** Cells were treated with vehicle (DMSO), saliva (2.4 μg protein/ml), LPS, or saliva + LPS for 18 h. **(A)** Saliva significantly reduced the relative expression levels of CCL5, soluble TNF Receptor I (sTNFRI), and TNF-α at 0.5 and 1 min exposure times when the cells were stimulated with LPS. **(B)** Image of blots exposed to film for 1 min corresponding to the treatments in **(A)**, cylinder = CCL5, oval = sTNFRI, and rectangle = TNF-α, (#) p < 0.05 when compared to vehicle control and when LPS was compared to LPS + saliva. Data are means ± SEM normalized to the vehicle treated array reported as relative expression levels determined by densitometry for 0.5 and 1 min exposure times.

**Figure 5:**
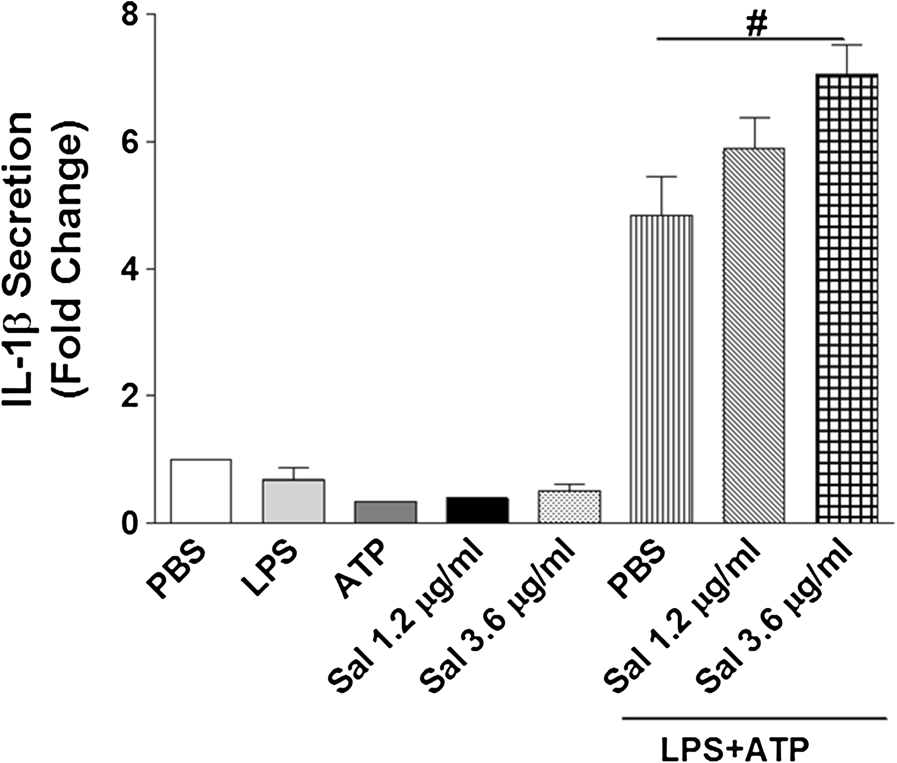
**Tick saliva increases IL-1β secretion by macrophages.** IL-1β is a pro-inflammatory cytokine produced by activated macrophages. Cells were treated with vehicle (PBS) or saliva (1.2 or 3.6 μg protein/ml) and then stimulated for 18 h with 0.76 μg/ml LPS. The cells were then pulsed with 5 mM ATP for 20 min and cultured for an additional 3 h. Surprisingly, saliva dose-dependently increased IL-1β secretion but significance (p < 0.05) was achieved at 3.6 μg protein/ml, (#) p < 0.05 when compared to vehicle. Data are means ± SEM, n = 3 assayed in duplicate.

Macrophages regulate the inflammatory and cellular immune responses by producing cytokines which influence the activity of lymphocytes. The pro-inflammatory cytokines tumor necrosis factor alpha (TNF-α), interleukin 6 (IL-6), IL-1 beta (IL-1β), and PGE_2_ are mediators of the inflammatory response [62]. In macrophages, PGE_2_ has been shown to have inhibitory effects on TNF-α and IL-12 production but enhances the production of IL-6 [41,42], which has both pro and anti-inflammatory effects. We used the RayBio® Mouse Cytokine Antibody Array to simultaneously test the effects of saliva on LPS-stimulated secretion of 22 cytokines and the role of PGE_2_ in any saliva-induced changes in cytokine secretion. Of the 22 cytokines tested, saliva only affected the secretion of Rantes (CCL5), TNF-α, and the soluble form of its receptor TNF Receptor I (sTNFRI). Saliva (2.4 μg protein/ml) significantly inhibited the relative expression levels of secreted pro-inflammatory cytokines CCL5 and TNF-α along with sTNFRI (Figure 3A and B). Since the cytokines secreted by macrophages are important to the inflammatory and immune responses, we used LPS to evaluate if saliva can decrease induced cytokine secretion. Saliva did inhibit LPS-stimulated secretion of these cytokines (Figure 4A and B). IL-1β is produced by activated macrophages, and this pro-inflammatory cytokine is an important mediator of the inflammatory response. However, using a mouse IL-1β ELISA Kit, we showed that saliva increased LPS-stimulated secretion of IL-1β by approximately 1.5 fold when compared to the vehicle control (Figure 5). Saliva had no significant effects on IL-6 and IL-12p40p70 cytokines which also regulate inflammation (Figures 3 and 4). The inhibitory effects of saliva on CCL5, sTNFRI, and TNF-α, were significantly reversed by AH 6809 (Figure 3A and B) which implicates the involvement of PGE_2_ in these effects.

### Tick saliva mimics PGE_2_-stimulated intracellular cAMP production

**Figure 6:**
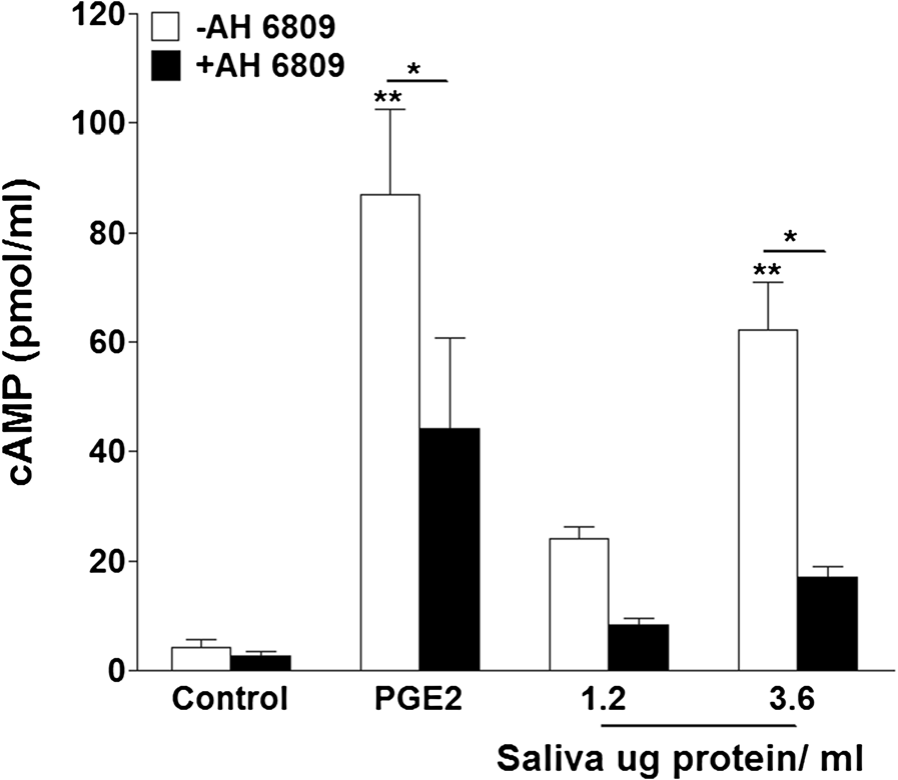
**Tick saliva increases intracellular cAMP levels in macrophages.** Cells were pretreated with AH 6809 (10 μM) for 15 min and challenged with vehicle (DMSO), saliva (1.2 and 3.6 μg protein/ml), or PGE_2_ (3 μM) for 7.5 min. Saliva increased macrophage intracellular cAMP production and signifcance (p < 0.05) was achieved at 3.6 μg protein/ml with stimulatory effects similar to that of PGE_2_, (**) p < 0.001 when compared to vehicle control. The stimulatory effects were reversed by AH 6809, (*) p < 0.01 when PGE_2_ was compared to PGE_2_ + AH 6809 and when saliva was compared to saliva + AH 6809. Data are means ± SEM, n = 3 assayed in duplicate.

When PGE_2_ binds to G protein-coupled receptors EP2/ EP4, the effects are mediated through increases in the second messenger cAMP. In macrophages, activation of PGE_2_ receptors are associated with increased migration [51] and inhibition of pro-inflammatory cytokines [41,42,63,64]. Therefore, we used a cAMP EIA assay to examine how the saliva-induced effects on macrophage migration and cytokine secretion correlate with changes in intracellular cAMP levels. After 7.5 min, saliva (3.6 μg protein/ml) and 3 μM PGE_2_ significantly increased cAMP production 62 ± 9 and 87 ± 16 pmol/ml respectively (Figure 6). The stimulatory effects of saliva and PGE_2_ were both substantially reversed by the receptor antagonist, which decreased cAMP concentrations to 45 ± 17 and 17 ± 2 pmol/ml respectively (Figure 6). This suggests that the PGE_2_ in tick saliva binds receptors EP2/ EP4 and mediates its effects through increases in intracellular cAMP production in the macrophages.

## Discussion

To sustain physical attachment for several days, ixodid ticks have evolved to produce saliva which contains biologically active molecules that modulate their host’s immune, inflammatory, hemostatic, and wound healing responses. We have previously shown that tick salivary constituent(s) have differential effects on the migratory and signaling activities of fibroblasts and macrophages [13-15]. Both cells are important in the wound healing cascade; however, macrophages are also key regulators of the inflammatory and immune responses. In wound healing, they phagocytose apoptotic neutrophils which limit their cytotoxic contents from spilling and damaging surrounding tissue [65,66]. Macrophages (M2) also trigger the proliferative phase of wound healing by secreting cytokines and growth factors such as PDGF which recruits fibroblasts to the site of injury [67]. The cytokines they produce control the inflammatory and cellular immune responses by influencing the activation and function of T lymphocytes. The lipid modulators they produce such as PGE_2_ also play a role in regulating these responses. Here, we examined the role of PGE_2_ in *D. variabilis* saliva on the regulation of macrophage and fibroblast migration, along with macrophage cytokine secretion by using the PGE_2_ receptor antagonist AH 6809.

It is well established that there is great similarity in the salivary components among different tick species. One consistency is the presence of prostaglandins [39,46-50], and particularly PGE_2_ in saliva, which has been shown to regulate dendritic cell differentiation, maturation, and cytokine production [31,32] and inhibit T lymphocyte proliferation [47]. Our results indicate that *D. variabilis* saliva like other ixodid tick species contains a high concentration of PGE_2_ and stimulates PGE_2_ secretion by macrophages. Therefore, ticks not only secrete components in their saliva to regulate host responses but also their salivary components induce cells to produce and secrete immuno-modulatory, anti-hemostatic, and anti-inflammatory effectors such as PGE_2_.

PGE_2_ has been shown to regulate the migratory activity of different cell types [51,54,68,69]. Therefore, it is logical that the PGE_2_ content in tick saliva is responsible for our previous observation of the saliva-induced regulation of macrophage [14] and fibroblast migration [13,15]. We show that the increased macrophage migration and decreased fibroblast migration are both sensitive to the PGE_2_ receptor antagonist AH 6809. These data are consistent with the studies showing differing effects of PGE_2_ on macrophage [51] and fibroblast [52-54] migration. In addition, fibroblasts treated with conditioned medium from saliva-treated macrophages had lower migratory rates, a response reversed by AH 6809. AH 6809 fully restored the migratory activity of fibroblasts treated with conditioned medium from saliva-treated macrophages, suggesting this response was mediated by PGE_2_. However, AH 6809 partially restored the migration of the cells treated with saliva only, meaning the saliva-induced inhibition was also regulated by some other salivary constituent(s). This is supported by our previous studies which have shown saliva treatment reduced migration in fibroblasts and cancer cells, a response that correlated with changes in downstream effectors of growth factor receptor signaling [13,15]. Our results demonstrating that saliva modulates migration in these cells are further substantiated by studies which identified changes in macrophage [70] and fibroblast [15] numbers at the feeding lesion.

Whether or not PGE_2_ is a pro or anti-inflammatory mediator is controversial [41,42,62,71,72]. Since it is imperative for ticks to control host responses, we believe the PGE_2_ in saliva dampens host inflammation. From our observation of 22 cytokines, we show saliva reduces the LPS-stimulated secretion of pro-inflammatory cytokines CCL5, TNF-α, and soluble TNF Receptor I (sTNFRI). CCL5 recruits macrophages, dendritic cells, basophils, eosinophils, mast cells, natural killer cells, and T lymphocytes to sites of inflammation and infection [73,74] where they either participate in resolving inflammation or provide cues for activation of the adaptive immune response. The decrease in CCL5 was reversed by the PGE_2_ receptor antagonist consistent with a report showing that tumor-secreted PGE_2_ inhibits CCL5 production in macrophages [63]. However, this reduction in CCL5 may be due to the chemokine binding protein, Evasin-4, which interacts with CCL5 and CCL11 and has been identified in tick SGE [19]. By decreasing CCL5, ticks can prevent macrophages from recruiting other leukocytes to the feeding lesion, therefore dampening the host inflammatory and immune responses. Chiefly produced by macrophages, TNF-α is a pleiotropic cytokine that serves as a key mediator of inflammation. It increases vascular permeability and cytokine production eliciting the recruitment of macrophages and neutrophils to sites of infection. In neutrophils, TNF-α has been shown to induce proliferation and apoptosis [75]. It can also induce blood clotting [76], therefore serving as a mechanism of containment during an infection. Low levels of TNF-α promote replacement or remodeling of damaged tissue by triggering fibroblast growth [77]. This cytokine can result in activation of an adaptive immune response since it contributes to the proliferative response in T lymphocytes [78]. However, the persistent presence of TNF-α can contribute to chronic inflammatory conditions as seen in rheumatoid arthritis (RA) [79]. We show that saliva reduces the secretion of TNF-α and its receptor in macrophages, and this effect was sensitive to the PGE_2_ receptor antagonist. This is supported by evidence indicating that in macrophages PGE_2_ works in concert with IL-6 to inhibit TNF-α production in a murine arthritis/lupus model [41]. Surprisingly, saliva did not affect the secretion of IL-6 and IL-12p40p70 or the anti-inflammatory cytokine IL-10 (data not shown). We expected saliva to impose some change on IL-6 secretion because in RA it is considered pro-inflammatory [41], and it is produced with TNF-α and IL-1β in other stress conditions [80]. Furthermore, both *in vitro*[81] and *in vivo*[80] studies have indicated the anti-inflammatory effects of IL-6. Since we have previously shown saliva increases the gene expression of anti-inflammatory cytokine IL-10 [14] which is indicative of an immune response shifted toward a T helper 2 phenotype [82], we anticipated saliva would increase the secretion of this cytokine but this effect was not observed (data not shown). Saliva did not reduce the IL-12 subunit IL-12p40p70 as we expected because PGE_2_ inhibits IL-12 production in macrophages [42] and production of this cytokine drives a pro-inflammatory response characterized as a T helper 1 reaction [83]. However, we are currently investigating the secretion of these cytokines at earlier time points as in our gene expression study in Kramer *et al*. [14]. In addition, we evaluated how saliva influences the secretion of the pro-inflammatory cytokine IL-1β. The production of this cytokine is tightly regulated by a multi- protein complex called an inflammasome. While saliva increases LPS-stimulated secretion of IL-1β, we have shown that the expression of the IL-1β receptor antagonist IL-1RN is also up-regulated and may serve as a countermeasure to any pro-inflammatory effects from this cytokine [14].

PGE_2_ modulates cellular activities via G protein-coupled receptors EP1-4 whose effects are mediated through calcium mobilization and cAMP production. In fibroblasts, PGE_2_ activation of EP2 and EP4 receptors leads to increases in cAMP production and inhibition of migration [52-54], comparable to our observation of saliva-induced decreases in fibroblast migration. We also observed the saliva-induced increases in cAMP production in macrophages correlated with the PGE_2_-mediated changes on migration and cytokine secretion. Using RAW264.7 macrophages, Tajima *et al*., [51] showed that PGE_2_ regulates LPS-stimulated migration through the EP4 receptor supporting our rationale for the PGE_2_ content in tick saliva as the modulator of macrophage migration. In our study we used the PGE_2_ receptor antagonist AH 6809 which binds EP2 but not EP4. However, we believe that the ability of AH 6809 to reverse the stimulatory effects of PGE_2_ and saliva on macrophage migration suggests that in IC-21 macrophages EP2 also plays a role. Also intracellular cAMP has been shown to have a central role in resolving inflammation [84,85]. The inhibitory effects of tumor-secreted PGE_2_ on macrophage CCL5 are mediated through cAMP [63] further supporting the idea that inhibitory effects of saliva on macrophage cytokine secretion are caused by PGE_2_ and mediated through cAMP.

## Conclusions

To facilitate the feeding process, ticks and other arthropods have evolved a repository of pharmacologically active molecules in their saliva to modulate the host’s inflammatory and immune responses. To our knowledge for the first time, our data illustrate that the saliva-induced changes on macrophage and fibroblast migration and cytokine secretion in macrophages are sensitive to a PGE_2_ receptor antagonist, suggesting these effects are mediated at least in part by PGE_2_ signaling through the second messenger cAMP. This indicates that the PGE_2_ content in tick saliva has roles in altering the migratory activity and cytokine profile of cells involved in inflammation and wound healing. These findings further demonstrate the complex nature of tick saliva and highlight the potential redundancy in the mechanisms utilized to regulate host responses.

## Abbreviations

SGE: Salivary gland extract; PDGF: Platelet-derived growth factor; PGE2: Prostaglandin E_2_; RANTES/CCL5: Regulated and normal T cell expressed and secreted; TNF-α: Tumor necrosis factor-alpha; sTNFRI: Soluble TNF receptor I; cAMP: Cyclic adenosine monophosphate; LPS: Lipopolysaccharide; TLR4: Toll-like receptor 4; HBSS: Hanks balanced salt solution; DMEM/F12: Dulbecco’s modified Eagle’s medium nutrient mixture F-12; FBS: Fetal bovine serum; DMSO: Dimethyl sulfoxide; TS: Tick saline; EIA: Enzyme immunoassay; PBS: Phosphate buffered saline; EP: E prostanoid; DP: D prostanoid; CM: Conditioned medium; IL: Interleukin; ELISA: Enzyme-linked immunosorbent assay; ATP: Adenosine triphosphate; cAMP: Cyclic adenosine monophosphate; SEM: Means ± standard errors of means; EP2: E prostanoid receptor 2; EP4: E prostanoid receptor 4; RA: Rheumatoid arthritis.

## Competing interests

The authors declare that they have no competing interest.

## Authors’ contributions

NP participated in the design of this study, carried out all experimental work, analyzed the data, and wrote the manuscript. GM participated in the cAMP assays. RS participated in the experimental design and provided intellectual support. LC provided intellectual support. JC assisted in data analysis and directed the project. All authors read and approved the final manuscript.
